# Graphene Nanosheet-Wrapped Mesoporous La_0.8_Ce_0.2_Fe_0.5_Mn_0.5_O_3_ Perovskite Oxide Composite for Improved Oxygen Reaction Electro-Kinetics and Li-O_2_ Battery Application

**DOI:** 10.3390/nano11041025

**Published:** 2021-04-16

**Authors:** Chelladurai Karuppiah, Chao-Nan Wei, Natarajan Karikalan, Zong-Han Wu, Balamurugan Thirumalraj, Li-Fan Hsu, Srinivasan Alagar, Shakkthivel Piraman, Tai-Feng Hung, Ying-Jeng Jame Li, Chun-Chen Yang

**Affiliations:** 1 Battery Research Center of Green Energy, Ming Chi University of Technology, New Taipei City 24301, Taiwan; smartindigoboy@gmail.com (Z.-H.W.); issyokenme@gmail.com (L.-F.H.); taifeng@mail.mcut.edu.tw (T.-F.H.); 2 Department of Chemical Engineering, Ming Chi University of Technology, New Taipei City 24301, Taiwan; xu04m4520@gmail.com (C.-N.W.); yjli@mail.mcut.edu.tw (Y.-J.J.L.); 3 Center of Precision Analysis and Material Research, National Taipei University of Technology, No. 1, Section 3, Chung-Hsiao East Road, Taipei 106, Taiwan; 12karikalan.n@gmail.com; 4 Department of Energy & Mineral Resources Engineering, Sejong University, Seoul 05006, Korea; balachem01@gmail.com; 5 Sustainable Energy and Smart Materials Research Lab, Department of Nanoscience and Technology, Alagappa University, Karaikudi 630002, India; sbn.chem@gmail.com (S.A.); apsakthivel@yahoo.com (S.P.); 6 Department of Chemical and Materials Engineering, Chang Gung University, Kwei-Shan, Taoyuan 333, Taiwan

**Keywords:** Ce-doped LaMnO_3_ perovskite, XPS of LaMnO_3_, bifunctional activity, probe sonication, carbon-based composite

## Abstract

A novel design and synthesis methodology is the most important consideration in the development of a superior electrocatalyst for improving the kinetics of oxygen electrode reactions, such as the oxygen reduction reaction (ORR) and the oxygen evolution reaction (OER) in Li-O_2_ battery application. Herein, we demonstrate a glycine-assisted hydrothermal and probe sonication method for the synthesis of a mesoporous spherical La_0.8_Ce_0.2_Fe_0.5_Mn_0.5_O_3_ perovskite particle and embedded graphene nanosheet (LCFM(8255)-gly/GNS) composite and evaluate its bifunctional ORR/OER kinetics in Li-O_2_ battery application. The physicochemical characterization confirms that the as-formed LCFM(8255)-gly perovskite catalyst has a highly crystalline structure and mesoporous morphology with a large specific surface area. The LCFM(8255)-gly/GNS composite hybrid structure exhibits an improved onset potential and high current density toward ORR/OER in both aqueous and non-aqueous electrolytes. The LCFM(8255)-gly/GNS composite cathode (ca. 8475 mAh g^−1^) delivers a higher discharge capacity than the La_0.5_Ce_0.5_Fe_0.5_Mn_0.5_O_3_-gly/GNS cathode (ca. 5796 mAh g^−1^) in a Li-O_2_ battery at a current density of 100 mA g^−1^. Our results revealed that the composite’s high electrochemical activity comes from the synergism of highly abundant oxygen vacancies and redox-active sites due to the Ce and Fe dopant in LaMnO_3_ and the excellent charge transfer characteristics of the graphene materials. The as-developed cathode catalyst performed appreciable cycle stability up to 55 cycles at a limited capacity of 1000 mAh g^−1^ based on conventional glass fiber separators.

## 1. Introduction

In recent decades, green or renewable energy system development has garnered great interest worldwide due to high energy demand and the desire to save the atmosphere from air pollution. Secondary batteries are often considered the best renewable energy resources and alternative energy systems for gasoline fuels to fulfill the energy demand with high energy density [1,2,3,4]. Among them, Li-O_2_ batteries have attracted much attention as the next-generation energy storage system with a high specific energy density that is integrated with low-cost and environmentally friendly air cathode catalysts. Their practical energy density is comparable to gasoline fuel and 10 times higher than commercially available Li-ion batteries [1,5,6]. Molecular oxygen (O_2_) spread throughout the atmosphere is mainly used as a renewable oxidant source for Li-O_2_ batteries. However, the practical viability of Li-O_2_ batteries is still challenging due to the poor rate of oxygen reaction kinetics (O_2_ reduction/evolution; ORR/OER), electrolyte instability and poor long-term stability [7,8,9,10]. On the other hand, noble metal catalysts such as Pt, Ru and Ir have been used as potential air electrodes in Li-O_2_ batteries. This is owing to their high catalytic activity toward ORR/OER; however, they are very expensive, low-abundance materials, and they deliver poor cyclability due to their poor bifunctional activity, which is also a further reason to limit the commercialization of Li-O_2_ batteries [6,11]. Therefore, non-precious metals/oxides and carbon-based material catalysts or their composites should be considered in the development of a new air electrode for Li-O_2_ battery application [12,13,14,15].

Perovskite oxides (ABO_3_) are the most frequently considered air catalysts in recently studied metal–air battery systems [7,16,17]. Because of their high electronic and ionic conducting properties, they have demonstrated excellent bifunctional catalytic activity towards ORR/OER reactions when the A sites and B sites are substituted with other elements [18,19]. Interestingly, lanthanum manganite (LaMnO_3_) perovskite is a well-known catalyst for ORR in the perovskite family due to its defective cation-deficient lattice and the presence of multiple oxidation states, such as Mn^3+^ and Mn^4+^ [20,21,22]. Conversely, it has a poorer OER activity than LaCoO_3_ and LaNiO_3_ due to the Mn-O bond’s lower binding energy on the LaMnO_3_ surface. However, the bifunctional ORR and OER properties of LaMnO_3_ can be tuned by the partial substitution of La and Mn sites with alkaline earth or rare-earth and transition-metal cations, respectively. Therefore, metal-ion-doped LaMnO_3_ perovskites have received enormous attention in Li-air or Li-O_2_ battery applications as cathode materials to replace the conventional Pt or Pt/C catalysts [23,24,25,26,27]. The discharge and recharge overpotentials of these LaMnO_3_-based cathodes are significantly reduced as compared to commercial carbon and Pt/C catalysts [28]. However, most of the LaMnO_3_-based perovskites are prepared by a sol–gel method, which produces non-porous microparticles with a low surface area, resulting in low storage capacity and poor cycle stability. Hence, the development of LaMnO_3_ perovskites with more porosity and a high surface area is still required to increase the oxygen and Li^+^ ion diffusion pathways for the high storage capacity and cyclability of Li-O_2_ batteries [25].

In this study, a catalyst based on cerium- (Ce) and iron (Fe)-co-doped LaMnO_3_ perovskite was prepared by the hydrothermal synthesis route. Glycine was used as a reducing and pore-generating agent in this process, resulting in mesoporous and large-surface-area perovskite material. Interestingly, the morphology of the particles was changed due to the addition of glycine. Moreover, the catalyst’s activity was improved by changing the mole ratio of Ce dopant in the La_1−x_Ce_x_Fe_0.5_Mn_0.5_O_3_ (x = 0.2 & 0.5 mol%) perovskite structure. To boost the electronic conductivity and electrocatalytic activity, the La_1−x_Ce_x_Fe_0.5_Mn_0.5_O_3_ perovskites were infused with graphene nanosheets (GNS) via a probe sonication method. This is an efficient way to combine the transition-metal oxides with GNS, known to be highly conductive mechanical support materials [5,6], enhancing the composite catalyst’s energy conversion and storage properties in scalable applications.

## 2. Experimental Section

### 2.1. Chemicals

Analytical-grade La(NO_3_)_3_∙6H_2_O and Ce(NO_3_)_3_∙6H_2_O (Alfa Aesar, Ward Hill, MA, USA), Fe(NO_3_)_3_∙9H_2_O (J.T. Baker, Center Valley, PA, USA), Mn(NO_3_)_2_∙4H_2_O (Alfa Aesar, Heysham, England), KOH (Acros Organics, Fair Lawn, NJ, USA), glycine and 5 wt.% Nafion solution (Sigma-Aldrich, Louis, MO, USA) were used as received. GNS powder was purchased from Scientech Corporation, Taiwan.

### 2.2. Preparation of Porous La_1−x_Ce_x_Fe_0.5_Mn_0.5_O_3_ Perovskite Oxides

**Scheme 1:**
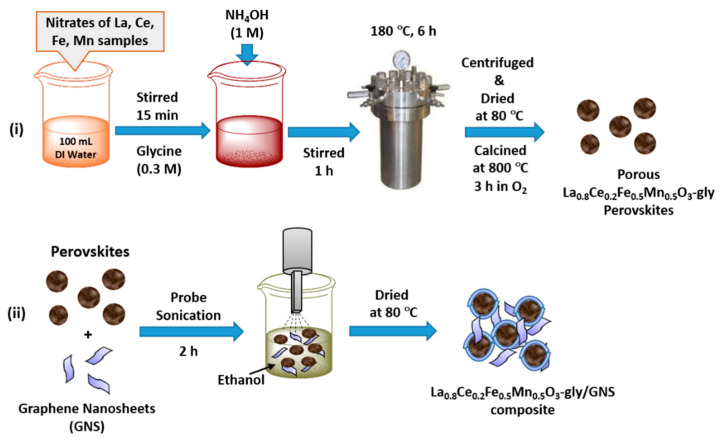
(**i**) Schematic for the synthesis of LCFM(8255)-gly perovskites. (**ii**) Schematic for the synthesis of LCFM(8255)-gly/GNS composite.

Porous La_1−x_Ce_x_Fe_0.3_Mn_0.7_O_3_ perovskite oxides were prepared via a hydrothermal synthesis method using a glycine–nitrate complex mixture as the precursor material. In brief, stoichiometric amounts of nitrates of La, Ce, Fe and Mn precursors were separately dissolved in de-ionized water (each in 50 mL) and mixed together with constant stirring by a magnetic stirrer for 15 min. Then, a 0.3 M aqueous glycine solution was added dropwise into the mixed precursor solution. The color of the solution changed from pale orange to red-orange, indicating the formation of a glycine–metal nitrate complex. Then, the final solution pH was adjusted to around 8.0–9.0 by 1 M NH_4_OH aqueous solution and stirred for 1 h at 25 °C. Finally, the mixed solution was poured into a 350 mL Teflon-lined autoclave for hydrothermal treatment at 180 °C for 6 h. After the temperature cooled down to 25 °C, the product was centrifuged and washed with deionized (DI) water and ethanol several times, and the collected residue was dried at 80 °C overnight. The dried powder was ground well, transferred into an alumina boat and kept inside a muffle furnace for calcination. The final powder was collected after calcining at 800 °C for 3 h in an air atmosphere, as depicted in Scheme 1i.

La_1−x_Ce_x_Fe_0.5_Mn_0.5_O_3_ perovskite oxide catalysts with different mole concentrations of Ce (x = 0.2 and 0.5) were synthesized in the presence of 0.3 M glycine (gly) to reduce the formation of bulk crystals of CeO_2_ and improve electrochemical performance. For comparison, La_0.5_Ce_0.5_Fe_0.5_Mn_0.5_O_3_ perovskite was also prepared by the same experimental procedure without the addition of glycine. Finally, the as-synthesized oxide catalyst samples, such as La_0.5_Ce_0.5_Fe_0.5_Mn_0.5_O_3_ and La_0.8_Ce_0.2_Fe_0.5_Mn_0.5_O_3_, were designated as LCFM(5555)-no gly, LCFM(5555)-gly and LCFM(8255)-gly, respectively.

### 2.3. Preparation of Porous LCFM(8255)-gly/GNS Composite

The probe sonication method was employed to prepare the GNS-wrapped LCFM(8255)-gly composite materials (designated as LCFM(8255)-gly/GNS). Briefly, 0.7 g of GNS powder was first dispersed in 100 mL ethanol and sonicated using a probe sonicator for 1 h. The probe sonicator was operated at 5 mV amplitude with a 20 min pulse-on and 5 min pulse-off procedure for 1 h. During the process, the probe sonicator’s output power and frequency were maintained at 2–4 W and 20 kHz, respectively. Then, about 0.3 g of the as-prepared LCFM(8255)-gly perovskite catalyst was added into the ethanolic GNS solution, followed by probe sonication for 1 h under the same conditions. Finally, the mixture was dried at 60 °C in an air oven; after this, the dried LCFM(8255)-gly/GNS composite catalyst could be used for further analysis, as depicted in Scheme 1ii. To compare with the electrochemical performance of LCFM(8255)-gly/GNS composite, the LCFM(5555)-gly/GNS composite catalyst was also prepared by the above-mentioned synthesis conditions.

### 2.4. Materials Characterization

Morphology and crystallinity of the synthesized materials were characterized by SEM (Hitachi-S2600, Hitachi Ltd., Tokyo, Japan) and XRD (Bruker D2 PHASER, Karlsruhe, Germany) analysis. TEM (JEM-2100, JEOL Ltd., Tokyo, Japan) equipment was used to confirm the morphology as well as the GNS layer on the perovskite microsphere particles. N_2_ adsorption–desorption isotherm analysis (Micromeritics, Gemini VII, Monchegladbach, Germany) was carried out to examine the materials’ surface area and porous nature. XPS (VG Scientific ESCALAB 250, Thermo Fisher, CA, USA) analysis was used to characterize the valence state of elements present in the composite samples. A three-electrode configuration for oxygen electrocatalysis study was employed using a CHI405 potentiostat (CHI Instruments, Austin, TX, USA) with a catalyst-modified rotating disk electrode (RDE) as working electrode, Ag/AgCl (sat. KCl) as reference electrode, and Pt wire as the counter electrode. Cyclic voltammetry (potential range = 2.0 to 4.5 V vs. Li/Li^+^; scan rate = 1 mV s^−1^) and AC impedance (frequency range = 1 MHz to 0.01 Hz; amplitude = 5 mV) analysis for two-electrode systems were performed using an Autolab PGSTAT30 (Metrohm Autolab B.V., Houten, The Netherlands).

### 2.5. Preparation of Electrode and Electrochemical Measurements

In order to prepare the oxygen electrode to evaluate the bifunctional activity of the oxide catalyst, the as-prepared composite catalyst (10 mg mL^−1^) was dispersed in a 1:3 *v*/*v* ratio DI water to isopropanol solvent mixture containing 80 µL of Nafion (5 wt.%) binder solution. This mixture was ultrasonicated (ultrasonic cleaner: DC300H; frequency −40 kHz; max. output power −300 W) for 1 h to develop a homogeneous catalyst ink. Then, about 20 µL of the catalyst ink was drop-coated onto the RDE (Geometric area = 0.196 cm^2^) surface and dried at 50 °C for 30 min. The as-modified RDE was used to further evaluate the bifunctional (ORR/OER) activity of the catalyst in a 0.1 M KOH electrolyte.

To prepare the oxygen cathode for Li-O_2_ battery application, a slurry was prepared by mixing the as-prepared catalyst samples, Ketjen black and PVDF in N-methyl pyrrolidone at a weight ratio of 80:10:10 and continuously stirred for 12 h at 25 °C to form a homogeneous mixture. Then, the catalyst mixture slurry was spray-coated (Sono-Tek Corporation, Milton, NY, USA) on both sides of the Ni-foam (2 × 2 cm^2^) matrix with a flow rate of 2 mL min^−1^ and dried at 120 °C for 12 h. Finally, the catalyst-coated Ni-foam electrodes were prepared as disks (diameter = 1.3 cm^2^) for cell assembly. The catalyst loading of each electrode was approximately 1 mg cm^−2^. EL-CELL was assembled in an Ar-filled glovebox environment (H_2_O < 0.5 ppm; O_2_ < 0.5 ppm) to evaluate the oxygen electrocatalyst’s performance toward Li-O_2_ battery application. Here, the as-prepared composite electrodes were used as a cathode, Li foil as an anode and commercial glass fiber (Whatman; thickness ~260 µm) as a separator for cell preparation. A 1 M Lithium bis(trifluoromethanesulfonyl) imide-Tetraethylene glycol dimethyl ether (LiTFSI-TEGDME)-based electrolyte was used with 0.5 M LiI as an additive. All the cells were tested at the potential cut-off range of 2.0–4.5 V vs. Li/Li^+^ at 100 mA g^−1^ using the Arbin BT2000 battery test system (Arbin Instruments, College Station, TX, USA). For all these cells, the oxygen supply was passed (10 mL min^−1^) through a thin microtube (diameter <1 mm) using pure O_2_ (99.999%) at 1 atm.

## 3. Results and Discussion

### 3.1. Surface and Structural Characterization of Perovskite Samples

**Figure 1:**
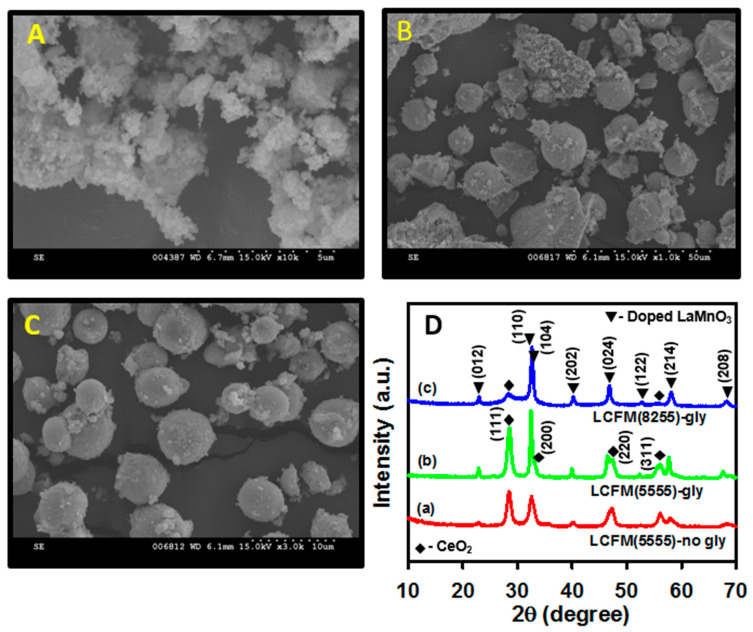
SEM images of (**A**) LCFM(5555)-no gly, (**B**) LCFM(5555)-gly and (**C**) LCFM(8255)-gly. (**D**) XRD patterns of (**a**) LCFM(5555)-no gly, (**b**) LCFM(5555)-gly and (**c**) LCFM(8255)-gly.

Glycine-assisted hydrothermal synthesis was performed to obtain the Ce- and Fe-co-doped LaMnO_3_ perovskites, such as LCFM(5555) and LCFM(8255), for energy conversion and storage application. The surface morphology of the as-prepared perovskite samples was investigated by SEM analysis, as shown in Figure 1A–C. Figure 1A exhibits the SEM image of LCFM(5555)-no gly, which reveals non-uniform particle growth during the hydrothermal reaction. Figure 1B shows the multi-shape morphology with a microscale that consists of LCFM(5555)-gly microspheres and CeO_2_ microplate-like structure. The microspheres of perovskite may form due to the efficient chelation chemistry between metal ions and the glycine metal–organic complex. These metal–organic frameworks can favor mesopore formation on the perovskite catalyst surface under the high-temperature calcination process. On the other hand, the high mol% of Ce doping can initiate the growth of aggregated CeO_2_ microstructures, resulting in lower electrochemical activity than the nanoparticles of CeO_2_. Therefore, we reduced the mol% of Ce to 0.2 to obtain the LCFM(8255) perovskite catalyst. Figure 1C clearly reveals the uniform LCFM(8255) microspheres with the co-existence of CeO_2_ nanoparticles. The crystallinity of these synthesized samples was also evaluated by XRD analysis. Figure 1D displays the XRD patterns of (a) LCFM(5555)-no gly, (b) LCFM(5555)-gly and (c) LCFM(8255)-gly. The planes are clearly indexed to partially doped LaMnO_3_ perovskite with rhombohedral crystal planes (JCPDS no. 89-8775) and a space group of R-3C. All three patterns showed the co-existence of cubic CeO_2_ phase (JCPDS no. 04-0593) with a significantly decreasing intensity of (111), (220) and (311) planes related to reducing the mol% of Ce doping. However, the addition of glycine can stabilize particle growth and improve the crystallinity of (b) LCFM(5555)-gly and (c) LCFM(8255)-gly samples, as compared to LCFM(5555)-no gly. In addition, the crystallite sizes of these samples were calculated using the Scherrer equation on the (110) plane; the derived values were 7.5, 16.7 and 10.1 nm for (a) LCFM(5555)-no gly, (b) LCFM(5555)-gly and (c) LCFM(8255)-gly, respectively.

**Figure 2:**
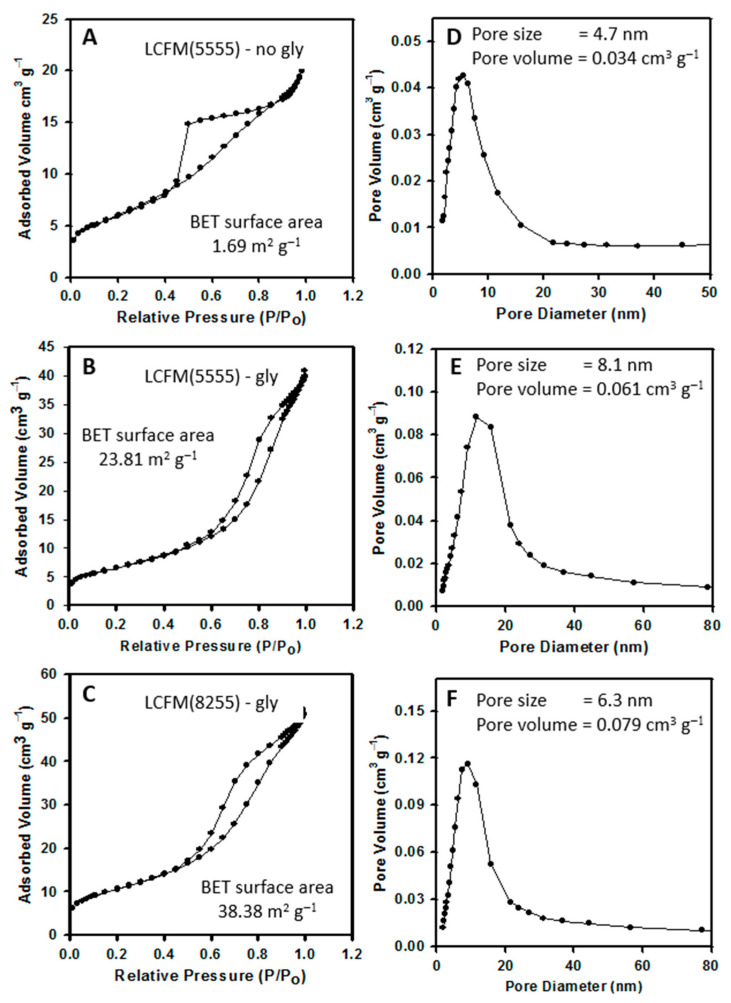
(**A**–**C**) N_2_ adsorption–desorption and (**D**–**F**) pore-size distribution curves of LCFM(5555)-no gly, LCFM(5555)-gly and LCFM(8255)-gly catalyst samples.

The surface area and pore-size distribution of the as-prepared perovskites were studied by using Brunauer–Emmett–Teller (BET) analysis. Figure 2A–C shows the N_2_ adsorption–desorption isotherm curves of LCFM(5555)-no gly, LCFM(5555)-gly and LCFM(8255)-gly samples. All the samples are followed the Type IV adsorption isotherm with an H_2_ hysteresis loop. The hysteresis loop at the relative pressure range from 0.4 to 1.0 P/P_o_ is attributed to the mesoporous nature of the sample. Hence, the LCFM(5555)-gly and LCFM(8255)-gly samples are exposed high specific surface areas of 23.81 and 38.38 m^2^ g^−1^, respectively, as compared to the LCFM(5555)-no gly (1.69 m^2^ g^−1^).

As seen in Figure 2A, a short-range interaction of the hysteresis loop was observed, and the desorption curve was suddenly dropped at 0.46 P/P_o_, which indicates that the lowest number of pores may occur in LCFM(5555)-no gly. It is also evidenced from the pore-size distribution curve of LCFM(5555)-no gly (Figure 2D), which shows the pore size and pore volume of about 4.7 nm and 0.034 m^3^ g^−1^, respectively. The pore size and pore volume are significantly enhanced to approximately 8.1 nm and 0.061 m^3^ g^−1^ for the LCFM(5555)-gly sample (Figure 2E), which is due to the multi-structure morphology of microspheres and microplates of LCFM(5555)-gly and CeO_2_, respectively. On the other hand, the pore size of LCFM(8255)-gly is reduced to 6.3 nm, and the pore volume is increased to approximately 0.071 m^3^ g^−1^, which confirms the residue of CeO_2_ nanoparticles may be positioned onto the pores of the LCFM(8255)-gly microsphere particles (Figure 2F). The obtained specific surface area (38.38 m^2^ g^−1^) of LCFM(8255)-gly perovskite is much higher than the previously reported LaMnO_3_ perovskites [16,23,24,25,29]. Thus, the existence of a large number of pores in LCFM(8255)-gly microspheres can provide support for the efficient diffusion of electrolyte ions and O_2_ gas in both O_2_ electrocatalysis and Li-O_2_ battery performance.

**Figure 3:**
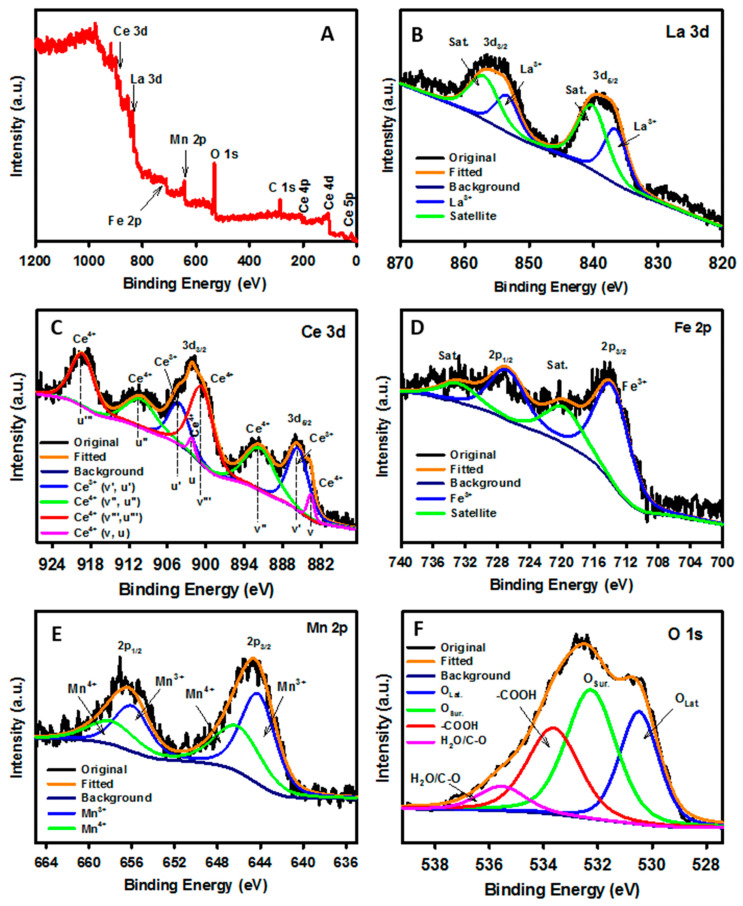
(**A**). XPS survey for elements present in LCFM(8255)-gly, deconvoluted XPS curves of (**B**) La 3d, (**C**) Ce 3d, (**D**) Fe 2p, (**E**) Mn 2p and (**F**) O 1s peak in LCFM(8255)-gly sample.

The XPS analysis further confirmed the presence of elements in the as-synthesized LCFM(8255)-gly perovskite catalyst. Figure 3A shows the wide-scan XPS curve of LCFM(8255)-gly perovskite sample, which confirmed the presence of La, Ce, Fe, Mn and O elements. The narrow-scan XPS curves of these elements were further fitted by the Gaussian–Lorentzian (G-L) method (30% of G-L ratio) by using XPSPEAK 4.1 software. Figure 3B shows the high-resolution La 3d XPS curve wherein the spin–orbit splitting (SOS) of 3d_5/2_ and 3d_3/2_ are separated with 16.8 eV. The multiplet at 3d_5/2_ is also separated by 4.2 eV, indicating the +3 oxidation state of lanthanum elements. In Figure 3C, eight peaks appeared for Ce 3d with an SOS of 18.6 eV. The doublet peaks at 885.6 eV (3d_5/2_) and 904.2 eV (3d_3/2_) belong to the Ce^3+^ state, whereas the other three doublet peaks at 883.7/902.3, 891.9/910.5 and 900.8/919.4 eV are related to the Ce^4+^ oxidation state [30]. The high-intensity peak at 919.4 eV also indicates the presence of CeO_2_ nanoparticles on the perovskite sample.

Figure 3D,E displays the deconvoluted spectra of Fe 2p and Mn 2p peaks, wherein the SOS of 2p_3/2_ and 2p_1/2_ are separated by 13.1 and 11.8 eV for Fe and Mn, respectively. The high-intensity peaks at 713.7 and 644.2 eV in Fe 2p_3/2_ and Mn 2p_3/2_ are corresponding to the +3 oxidation state of Fe and Mn elements in the LCFM(8255)-gly sample. In Mn 2p_3/2_, two doublet peaks were observed for the co-existence of the mixed oxidation state of Mn^3+^ and Mn^4+^. Several Mn^3+^ can be oxidized into Mn^4+^ while doping with other elements on the B site; however, the high intensity of Mn^3+^ indicates that the main component is present in the form of +3 in composite [27]. The ratio of Mn^4+^/Mn^3+^ was calculated as 0.68, and it was obtained from the peak area of Mn^4+^ and Mn^3+^. Furthermore, the core-level spectrum of O 1s peak (Figure 3F) is deconvoluted into four peaks wherein the peaks at 530.4 and 532.2 eV are corresponding to the lattice O_lat._ (O^2−^) and surface-level adsorbed oxygen species O_sur._ (O^−^, O_2_^−^ or O_2_^2−^). The ratio of O_lat._/O_total_ is approximately 0.27, which confirms the presence of oxygen vacancies. Moreover, the high intensity of the O_sur._ peak indicates the stronger covalence of the B-O bond, which favors the O_2_^−^/OH^−^ exchange. The obtained results suggest that the doping of Ce and Fe in LaMnO_3_ perovskite structures can increase the Mn^4+^ generation and oxygen vacancies, which will improve the kinetics of ORR and OER performance [25,26,27].

### 3.2. Surface and Structural Characterization of LCFM(8255)-gly/GNS Composite Catalyst

**Figure 4:**
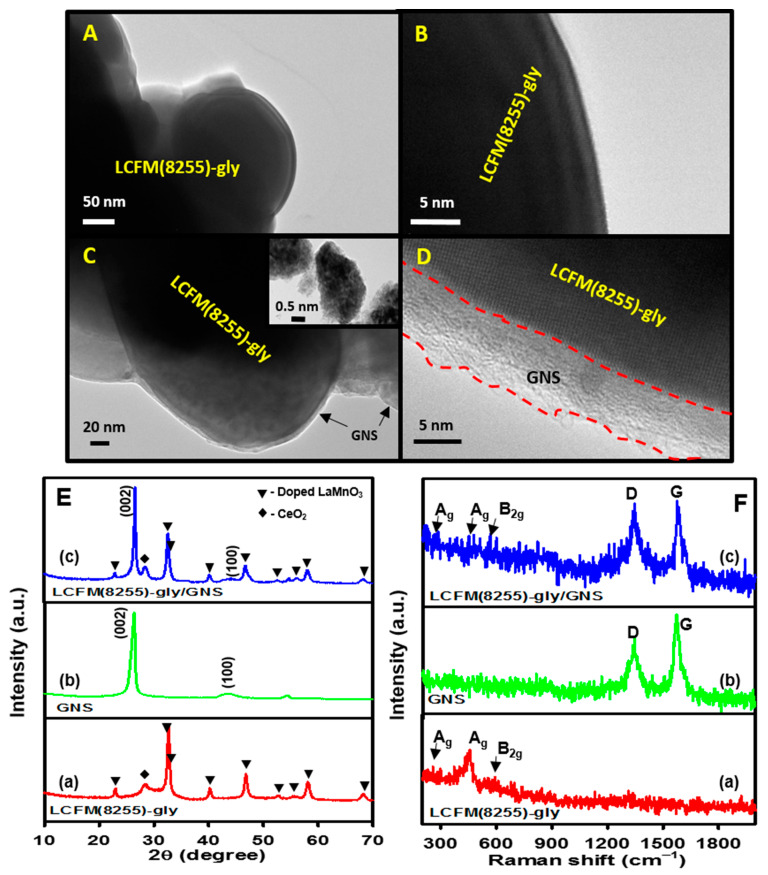
(**A**,**B**) TEM images of LCFM(8255)-gly and (**C**,**D**) LCFM(8255)-gly/GNS composite. (**E**) XRD patterns and (**F**) micro-Raman spectra of (**a**) LCFM(8255)-gly, (**b**) GNS and (**c**) LCFM(8255)-gly/GNS composite.

In this work, the as-synthesized LCFM(8255)-gly microsphere sample was combined with GNS to improve the electrochemical properties. The composite catalyst was already denoted as LCFM(8255)-gly/GNS. The integration of GNS with LCFM(8255)-gly microspheres is clearly characterized by TEM, XRD and micro-Raman spectroscopy analyses. Figure 4A–D displays the TEM images of LCFM(8255)-gly and LCFM(8255)-gly/GNS composite catalysts. The inset of Figure 4C clearly reveals the decoration of GNS on the LCFM(8255)-gly particles, on which the graphene sheets completely cover the perovskite particles (Figure 4C,D); however, it is not found in the other catalysts presented in Figure 4A,B. The average thickness of the GNS layer on LCFM(8255)-gly particles is ca. 6.5 nm.

Figure 4E depicts the XRD patterns of (a) LCFM(8255)-gly, (b) GNS and (c) LCFM(8255)-gly/GNS composite catalysts, which confirms the existence of (002) and (100) planes of GNS in the composite sample. The crystallinity of LCFM(8255)-gly sample was clearly discussed in the previous section. However, the diffraction peak of the (002) plane of GNS has shifted to a lower angle in the LCFM(8255)-gly/GNS composite sample, which indicates the disorder of graphene sheets by the interaction with LCFM(8255)-gly particles. Moreover, the disordered properties are further confirmed by micro-Raman spectra (Figure 4F). The typical D band and G band of the GNS appeared around 1347 and 1574 cm^−1^ (Figure 4F(b)), whereas it appeared around 1347 and 1576 cm^−1^ for the LCFM(8255)-gly/GNS composite (Figure 4F(c)). The D/G intensity ratios of GNS and LCFM(8255)-gly/GNS composite catalysts are calculated to ca. 0.98 and 1.0, respectively. These results indicate that the disordered graphene layer is possible in a composite sample and that the basal plane of GNS can be attached strongly to the perovskite microsphere particles that renders more uniform coverage on the surface of the particle. In addition, our micro-Raman spectra also reveal that the presence of LCFM(8255)-gly particles by the appearance of peaks at 264, 476 and 592 cm^−1^ are related to the rotational (A_g_) and Jahn–Teller stretching (A_g_, B_2g_) modes of the rhombohedrally distorted LaMnO_3_ perovskite, respectively [31]. The above results demonstrate clear evidence of the formation of LCFM(8255)-gly/GNS composite catalyst.

### 3.3. Electrochemical Performances

#### 3.3.1. Oxygen Electrocatalysis

**Figure 5:**
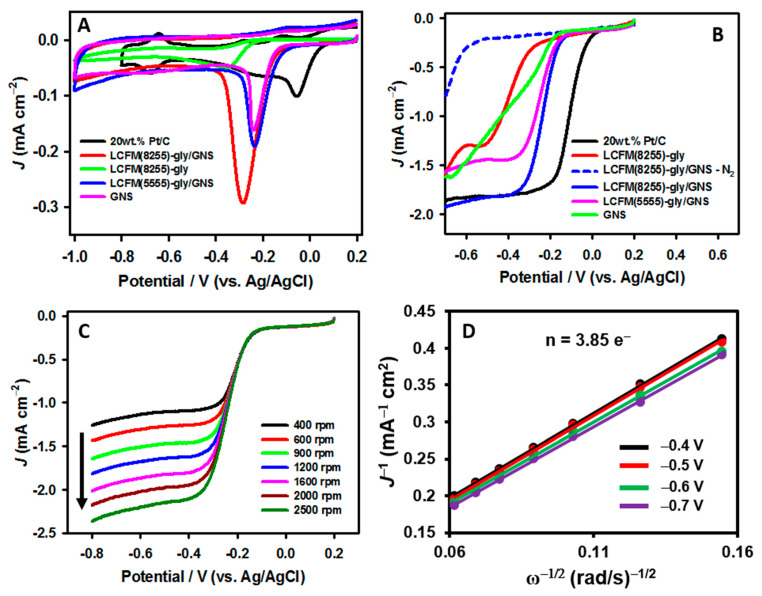
(**A**). CVs and (**B**). LSVs for ORR kinetics at various electrodes recorded in O_2_-saturated 0.1 M KOH; Scan rate: 20 mV s^−1^; Rotation speed: 1600 rpm for LSV measurements. (**C**). LSVs of RDE measurements using LCFM(8255)-gly/GNS composite electrode at various rotation speeds from 400 to 2500 rpm in O_2_-saturated 0.1 M KOH; Scan rate: 20 mV s^−1^, (**D**). Koutecky–Levich plot of j^−1^ vs. ω^−1/2^.

The electrocatalytic ORR studies were performed by cyclic voltammetry (CV) and linear sweep voltammetry (LSV) in 0.1 M KOH electrolyte using different electrodes; the data are displayed in Figure 5A,B. Typical ORR curves were observed from CV scans (20 mV s^−1^) for all the catalyst-modified electrodes, such as 20 wt.% of Pt/C, GNS, LCFM(8255)-gly, LCFM(5555)-gly/GNS and LCFM(8255)-gly/GNS composite catalysts (Figure 5A). The ORR peak potential (at −0.35 V vs. Ag/AgCl) of LCFM(8255)-gly perovskite is far better than the previously published undoped LaMnO_3_ perovskite catalyst (at −0.48 V vs. Ag/AgCl) [29]. However, the peak potential of the composite electrodes, i.e., LCFM(5555)-gly/GNS and LCFM(8255)-gly/GNS, was significantly improved, nearly to commercial Pt/C electrode activity, which is due to the high electrical conductivity of GNS. Further, the LSV polarization studies were conducted for the above-mentioned electrodes by the RDE measurements, which operated at a 1600 rpm rotation speed and a 20 mV s^−1^ scan rate in O_2_-saturated 0.1 M KOH electrolyte. The LCFM(8255)-gly/GNS composite electrode shows good electrocatalytic activity to the reduction of oxygen molecule, as can be observed in Figure 5B, in the presence of N_2_- and O_2_-saturated electrolytes. In addition, the LCFM(8255)-gly/GNS composite electrode (at −0.12 V vs. Ag/AgCl; 1.82 mA cm^−2^) shows higher catalytic activity with respect to the onset potential and limiting current density than that of LCFM(8255)-gly (at −0.26 V vs. Ag/AgCl; 1.11 mA cm^−2^), GNS (at −0.17 V vs. Ag/AgCl; 1.62 mA cm^−2^) and LCFM(5555)-gly/GNS composite electrodes (at −0.14 V vs. Ag/AgCl; 1.43 mA cm^−2^). The obtained results are almost closer to the activity of commercial 20 wt.% Pt/C catalyst (at 0.01 V vs. Ag/AgCl; 1.77 mA cm^−2^). The half-wave potentials of 20 wt.% Pt/C and LCFM(8255)-gly/GNS composite electrodes are −0.106 and −0.227 V vs. Ag/AgCl, respectively, and the potential difference (ΔE_1/2_ = E_1/2,catalyst_ − E_1/2,Pt/C_) is ca. 121 mV. In addition, the RDE measurements were conducted using the LCFM(8255)-gly/GNS composite electrode at different rotational speeds in the range of 400–2500 rpm (Figure 5C). The electron transfer number (n) can be calculated from these RDE polarization curves, which is one of the major factors for ORR catalyst evaluation [25,27]. To analysis the ORR reaction kinetics, the Koutecky–Levich Equations (1) and (2) can be followed based on the relation of inverted limiting current density J^−1^ versus square roots of rotation speed (ω^−1/2^), the data can be seen in Figure 5D.

(1) $$\frac{1}{J}=\frac{1}{J_{L}}+\frac{1}{J_{K}}=\frac{1}{B\omega^{1/2}}+\frac{1}{J_{K}}$$

(2) $$B=0.62nFC_{0}\;D_{0}^{1/2}v^{-1/6}$$

where *J*, *J_L_* and *J_K_* are the measured currents, diffusion-limited current and kinetic current densities, respectively, *ω* is the electrode angular rotation speed, *n* is the electron transfer number, *F* is the Faraday constant (96485 A∙s mol^−1^), *ν* is the kinematic viscosity (0.01 cm^2^ s^−1^), *C*_0_ is the bulk concentration (1.9 × 10^−5^ mol cm^−3^) and *D_0_* is the diffusion coefficient (1.2 × 10^−6^ mol cm^−3^) of dissolved O_2_ in 0.1 M KOH electrolyte. From the slope of the linear fit in Figure 5D, the average electron transfer number of the LCFM(8255)-gly/GNS composite catalyst is calculated as ca. 3.85, which indicates the one-pot, four-electron transfer pathway. The reaction mechanism for the four-electron transfer of the ORR process can be expressed as follows [25]:

(3) $$O_{2}+H_{2}O+4e^{-}\to 4{\mathrm{OH}}^{-}$$

The above-obtained results indicate that the LCFM(8255)-gly/GNS composite sample is the best ORR active catalyst and a better replacement to the high-cost conventional Pt/C in fuel cells and metal–air batteries.

#### 3.3.2. Bifunctional ORR/OER Activity

**Figure 6:**
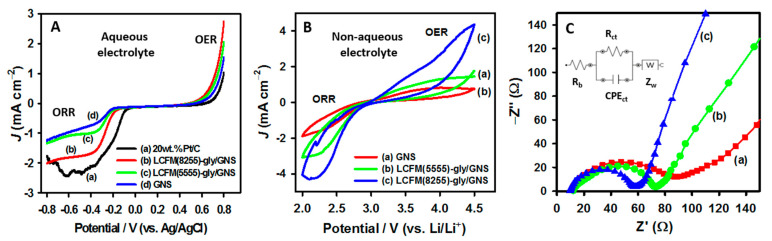
(**A**). LSVs for ORR and OER kinetics at various electrodes recorded in O_2_-saturated 0.1 M KOH; Scan rate: 20 mV s^−1^; Rotation speed: 1600 rpm, (**B**). CVs for ORR and OER kinetics using (**a**) GNS, (**b**) LCFM(5555)-gly/GNS and (**c**) LCFM(8255)-gly/GNS-based Li-O_2_ cells in 1 M LiTFSI-TEGDME + 0.5 M LiI at a scan rate of 1 mV s^−1^, (**C**). EIS data for electron transfer properties of (**a**) GNS, (**b**) LCFM(5555)-gly/GNS and (**c**) LCFM(8255)-gly/GNS-based Li-O_2_ cells.

To further evaluate the bifunctional characteristics, LSV polarization curves were obtained using (a) 20 wt.% Pt/C, (b) LCFM(8255)-gly/GNS, (c) LCFM(5555)-gly/GNS and (d). GNS electrodes in O_2_-saturated 0.1 M KOH electrolyte (Figure 6A). The total overpotential difference (ΔE = E_OER_ − E_ORR_) between ORR and OER curves of those electrodes was measured at 0.5 and 1 mA cm^−2^, respectively. The ΔE value of LCFM(8255)-gly/GNS composite electrode is ca. 0.94 V vs. Ag/AgCl, which is very close to the 20 wt.% Pt/C (at 0.92 V vs. Ag/AgCl) and lower than the LCFM(5555)-gly/GNS (at 1.02 V vs. Ag/AgCl) and bare GNS (at 1.06 V vs. Ag/AgCl) electrodes. Hence, the results revealed that the LCFM(8255)-gly/GNS composite electrode is a superior bifunctional catalyst, which can be used as a cathode in Li-O_2_ battery application instead of a conventional Pt/C catalyst and other carbon-based cathodes.

Prior to use these catalysts as Li-O_2_ battery cathode, their bifunctionality is to be further examined in a non-aqueous electrolyte (1 M LiTFSI-TEGDME + 0.5 M LiI) system. Figure 6B shows the CVs of ORR and OER activity of (a) GNS, (b) LCFM(5555)-gly/GNS, and (c) LCFM(8255)-gly/GNS-based cathodes in O_2_-saturated non-aqueous electrolyte at a scan rate of 1 mV s^−1^. It is clearly indicated that the LCFM(8255)-gly/GNS composite cathode shows much lower onset overpotential and high peak current density than the GNS and LCFM(5555)-gly/GNS electrodes. This superior ORR and OER activity can be achieved due to the intrinsic electronic conductivity of LCFM(8255)-gly/GNS composite catalyst materials. The Nyquist plot (Figure 6C) of EIS data were taken to evaluate the charge transfer properties of the (a) GNS, (b) LCFM(5555)-gly/GNS and (c) LCFM(8255)-gly/GNS cathodes. The inset of Figure 6C shows the equivalent-circuit model for the fitting of the Nyquist plots. It was found that the lower charge transfer impedance can be observed at LCFM(8255)-gly/GNS cathodes, as compared to GNS and LCFM(5555)-gly/GNS. As noted, the charge transfer characteristics of perovskite and graphene composite are significantly improved due to the interaction between the redox-active perovskite structure and conductive graphene support.

#### 3.3.3. Li-O_2_ Battery Performance

**Figure 7:**
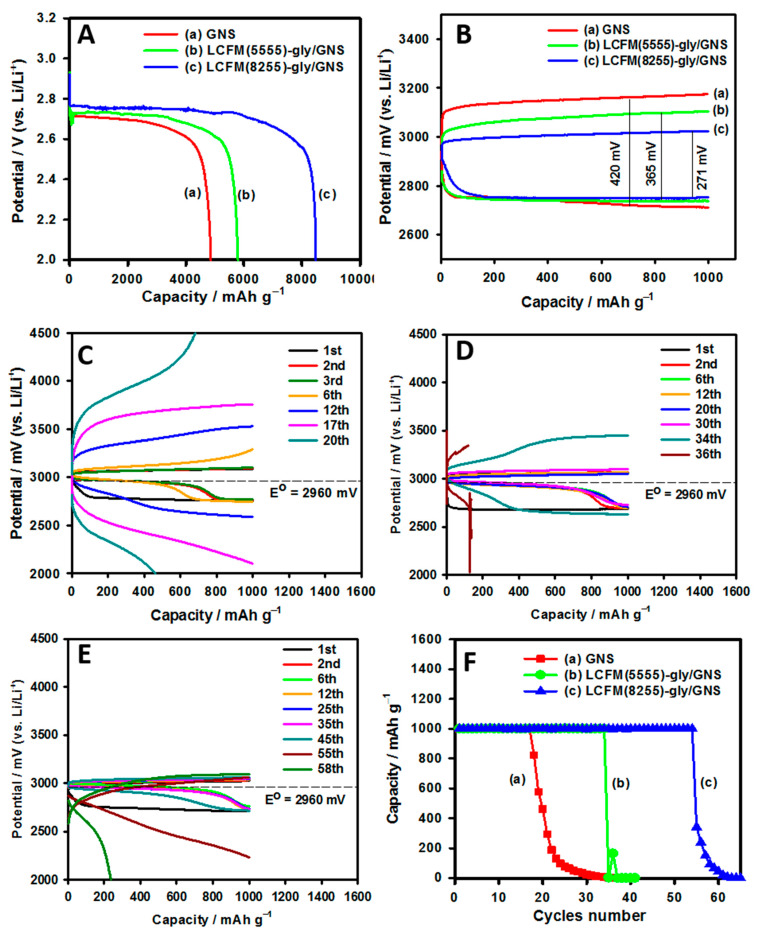
(**A**) Initial discharge curves of (**a**) GNS, (**b**) LCFM(5555)-gly/GNS and (**c**) LCFM(8255)-gly/GNS-based Li-O_2_ cells at a current density of 100 mA g^−1^. Discharge–charge curves of (**a**) GNS, (**b**) LCFM(5555)-gly/GNS and (**c**) LCFM(8255)-gly/GNS-based Li-O_2_ cells for (**B**) overpotential difference and (**C**–**E**) cycling stability analysis. (**F**) Cycle life of (**a**) GNS, (**b**) LCFM(5555)-gly/GNS and (**c**) LCFM(8255)-gly/GNS-based Li-O_2_ cells. Current density: 100 mA g^−1^; Limited discharge capacity: 1000 mAh g^−1^; Electrolyte: 1 M LiTFSI-TEGDME + 0.5 M LiI.

Figure 7A shows the discharge (charge storage) curves of (a) GNS, (b) LCFM(5555)-gly/GNS and (c) LCFM(8255)-gly/GNS-composite-based Li-O_2_ battery cathodes, operated in the voltage range of 2000–4500 mV vs. Li/Li^+^ at a current density of 100 mA g^−1^, which shows capacities around 4860, 5796 and 8475 mAh g^−1^, respectively. The discharge–charge overpotential difference of the cathodes was also studied at the limiting discharge–charge capacity of 1000 mAh g^−1^ at a current density of 100 mA g^−1^. As clearly seen in Figure 7B, the LCFM(8255)-gly/GNS composite cathode shows a very low overpotential difference of about 271 mV vs. Li/Li^+^, as compared to LCFM(5555)-gly/GNS (around 365 mV vs. Li/Li^+^) and GNS cathodes (around 420 mV vs. Li/Li^+^). The obtained results are in strong accordance with the CV and RDE measurements. To further evaluate the performance of Li-O_2_ batteries, the cycle stability curves of the as-developed cathodes were obtained at 100 mA g^−1^ with limiting capacities of 1000 mAh g^−1^; the data can be seen in Figure 7 ((C) GNS, (D) LCFM(5555)-gly/GNS and (E) LCFM(8255)-gly/GNS samples). The comparison of cycle stability with respect to discharge-limiting capacity was also shown in Figure 7D. As noted in cycle stability curves, the discharge–charge polarization overpotential rapidly increases to the high cut-off voltage for GNS (up to only 20 cycles) rather than composite cathodes, even in the presence of LiI redox mediator in the electrolyte system, confirms the poor, sluggish ORR/OER kinetics. This is also due to the parasitic reaction between the electrolyte ions and carbon cathodes. Conversely, the LCFM(5555)-gly/GNS composite cathode exhibits stable polarization for up to 35 cycles, and the charge potential is below 3500 mV vs. Li/Li^+^; however, the discharge overpotential reached cut-off voltage. Therefore, a sudden drop in discharge capacity after 35 cycles was observed (Figure 7C), due to the less porous nature of LCFM(5555)-gly particles. On the other hand, the battery with LCFM(8255)-gly/GNS cathode also shows stable polarization up to 55 cycles, as the charge potential does not increase beyond 3100 mV vs. Li/Li^+^, indicating the excellent catalytic activity of the LCFM(8255)-gly/GNS composite. The charge potential on both composite catalysts is still lower upon increasing the cycle life of the battery; this is mainly due to the LiI-soluble catalyst, which can also help to decompose the Li_2_O_2_ discharge product during the charging process. On the other hand, the increasing discharge potential range towards the cut-off discharge voltage is due to the blocking of oxygen and Li^+^ ion diffusion sites by the continuous deposition of Li_2_O_2_ during the discharge process, resulting in poor ORR activity of the catalyst in prolonged cycles. Thus, the results suggest that the air cathode should have a large number of pores and high surface area catalysts, which may enhance the oxygen and Li^+^ ion diffusion property that facilitates the high-performance battery lifespan. Therefore, as compared to previous reports [23,24,25,26,27] demonstrating the perovskite oxide catalyst prepared by citric acid and urea as fuel or a chelating agent, the present synthesis method with glycine can increase the surface area due to the presence of a large volume of mesopores and the integration of conductive GNS as a support, which can further improve the electrocatalytic properties of the perovskite materials.

## 4. Conclusions

A simple synthesis methodology has been developed for synthesizing a highly porous, high-surface-area A-site- and B-site-doped LaMnO_3_ perovskite. XRD analysis confirmed the distorted rhombohedral crystal structure for this perovskite is due to Ce^3+^ and Fe^3+^ cation doping, respectively. The CV results confirmed that the LCFM(8255)-gly/GNS composite electrode shows better ORR onset potential and peak current density than the bare LCFM(8255)-gly and LCFM(5555)-gly/GNS composite electrodes. The electrochemical ORR/OER kinetics of the proposed catalyst can be significantly improved by incorporating conductive GNS as support and reducing the mol% of Ce ion doping on the A site. Furthermore, the effect of dual cation doping on the LaMnO_3_ structure increases the ratio of Mn^4+^/Mn^3+^ species and generates more oxygen vacancies on the perovskite crystal structure. Finally, the as-developed LCFM(8255)-gly/GNS composite catalyst achieved efficient bifunctional activity comparable to commercial Pt/C catalysts. Therefore, the proposed synthesis method can derive low-cost and highly active perovskite materials, including a large-surface-area and highly porous structure, as the air cathode for energy storage and conversion applications.

## Data Availability

No new data were created or analyzed in this study.
